# Mutations and Phylogenetic Analyses of SARS-CoV-2 Among Imported COVID-19 From Abroad in Nanjing, China

**DOI:** 10.3389/fmicb.2022.851323

**Published:** 2022-03-17

**Authors:** Ning Zhao, Nan Zhou, Huafeng Fan, Jie Ding, Xingyu Xu, Xiaoqing Dong, Xiaoxiao Dong, Dandan Xu, Xiaoyu Min, Yan Yu, Hongjin Gong, Lingfeng Mao, Min He

**Affiliations:** ^1^Microbiology Laboratory, Nanjing Municipal Center for Disease Control and Prevention, Nanjing, China; ^2^Hangzhou Baocheng Biotechnology Co., Ltd., Hangzhou, China

**Keywords:** SARS-CoV-2, genomes, variant, phylogeny, evolution

## Abstract

Coronavirus disease 2019 (COVID-19), caused by severe acute respiratory syndrome coronavirus 2 (SARS-CoV-2), has become a pandemic and is threatening human health globally. The rapid genome sequencing and bioinformatic analysis of SARS-CoV-2 have become a helpful tool in the battle against the COVID-19. Here, we report the genetic characteristics, variations and phylogenetic analysis of SARS-CoV-2 sequenced from 42 clinical specimens. The complete genomes sequencing of SARS-CoV-2 were performed using Oxford Nanopore sequencing. All genomes accumulated mutations compared to the Wuhan-Hu-1 (GenBank Accession No: MN908947.3). Our data of the 42 whole genomes revealed 16 different lineages. The B.1.1 lineage was the most frequent, and 5, 2, 2, 3, and 1 sequences were classified as lineages of B.1.1.7, B.1.351, P.1, B.1.617.2, and C.37, respectively. A total of 328 nucleotide mutation sites were found in 42 genomes, among which A23403G mutation (D614G amino acid change in the spike protein) was the most common substitution. The phylogenetic trees of 42 SARS-CoV-2 sequences and GISAID-available SARS-CoV-2 sequences were constructed and its taxonomic status was supported. These results will provide scientific basis for tracing the source and prevention and control of SARS-CoV-2 imported from abroad in Nanjing, China.

## Introduction

The Coronavirus disease 2019 (COVID-19) is infection caused by the severe acute respiratory syndrome coronavirus 2 (SARS-CoV-2). Since it was first reported to the World Health Organization (WHO) in December 2019, severe acute respiratory syndrome coronavirus 2 (SARS-CoV-2) has spread to more than 200 countries and territories, causing 276,436,619 confirmed infections, including 5,374,744 deaths worldwide (23 December 2021, daily online worldwide data about COVID-19^1^). In the initial stage of infection, the infected person could be asymptomatic, while some experience mild to moderate symptoms, such as headache, cough, high fever, or pneumonia in severe cases (Koyama et al., 2020). According to the data available, infection is usually mild in healthy adults, while mortality is higher in individuals of advanced ages and the ones with comorbidities (Bonanad et al., 2020; Ruan, 2020; Yanez et al., 2020). Meanwhile, fatality rates vary widely among countries. This might be due to different demographics structure, adherence to public health guidelines such as wearing masks, frequent hand washing and use of disinfectants, as well as measures such as isolation, partial or total blockade in different countries to curtail viral transmission (Omotoso et al., 2020).

SARS-CoV-2 is a novel member of the coronavirus family, belonging to the genus *Betacoronavirus* (Zhu et al., 2020), and includes four basic proteins, spike (S), envelope (E), membrane (M), and nucleocapsid (N) proteins. Although related viruses have been found in bats and pangolins, the origin of the outbreak remains unclear. Compared with Severe Acute Respiratory Syndrome coronavirus (SARS-CoV) and Middle East Respiratory Syndrome coronavirus (MERS-CoV), SARS-CoV-2 is more pathogenic (Hossain et al., 2021). It has been reported that 2 months after the virus was first reported, more than 100 polymorphic sites have been identified in the SARS-CoV-2 protein coding region (Lu et al., 2020b; Ralph et al., 2020). At the same time, SARS-CoV-2 is undergoing recombination, which is common in RNA viruses (Yi, 2020). The genome of SARS-CoV-2 is evolving (Zhu et al., 2021). Following the outbreak of the COVID-19 pandemic, several SARS-CoV-2 variants, such as lineage B.1.1.7, B.1.351 and so on, have emerged and are now spreading globally. The emergence of variants that posed a greater risk to global public health in late 2020 prompted the characterization of specific Variants of Concern (VOCs) and Variants of Interest (VOIs) to prioritize global surveillance and research, and ultimately inform the current response to the COVID-19 pandemic. Currently, VOCs includes Pango lineages B.1.1.7 (Alpha), B.1.351 (Beta), P.1 (Gamma), B.1.617.2 (Delta) and B.1.1.529 (Omicron), while VOIs includes Pango lineages C.37 (Lambda) and B.1.621 (Mu). Although the pathogenesis of the disease is not fully elucidated, the increased transmissibility of these variants suggests that variation in virus strains also appears to be associated with differences in transmission/infectivity and/or severity (Villoutreix et al., 2021). These variants alter infectivity, disease severity, or interaction with host immunity, highlighting the importance of genomic surveillance (Márquez et al., 2021). Global transmission patterns and genomic diversity are critical to elucidating the dynamics of a pandemic. Comprehensive genomic diversity analysis of virus sequences from different countries will provide insight into the global transmission pattern, virulence and pathogenesis of SARS-CoV-2.

Since the SARS outbreak in 2002–2003, genomic information has become increasingly important in responding to outbreaks caused by pathogenic coronavirus (Llanes et al., 2020). The publication of the complete genome sequences of SARS-CoV-2 enabled the development of RT-PCR assays for the detection of SARS-CoV-2 early in the spread of the epidemic and to serve as a diagnostic criterion during the ongoing COVID-19 pandemic (Kasterena et al., 2020; Wu et al., 2020). Meanwhile, the whole genomes data of SARS-CoV-2 can be used as a supplement to the routine diagnostic tests. Genome sequencing is an effective way to understand the viruses and reveal a variety of information. The combination of genomic information with epidemiological data can determine the transmission network of the virus and infer its potential origin (Fauver et al., 2020; Lu et al., 2020a; Rockett et al., 2020). By comparing multiple genomes, it is possible to study the phylogenetic relationships of viruses, transmission patterns, evolutionary rates and the role of mutations in infection and disease severity, and vaccine development (Llanes et al., 2020). In view of the importance of viral genomic information, a rapid method to obtain the SARS-CoV-2 genome is needed. With the continuous development of sequencing technology, Oxford-Nanopore sequencing has become one of the powerful means for the rapid detection of pathogens (Yan et al., 2021). Oxford Nanopore Technologies (ONT) device with long-read genome sequencing, is portable, inexpensive and requires minimal laboratory infrastructure or technical expertise for sample preparation, allowing for flexible rapid sequencing analysis (Jain et al., 2016). Nanopore sequencing provides an alternative to the existing virus WGS short reading platforms and has many advantages. Bull et al. (2020) demonstrated the accurate consensus-level SARS-CoV-2 sequence determination with ONT data. The suitability of ONT sequencing in standard phylogenetic analysis was confirmed. The ONT device has previously been used to monitor viruses during outbreaks of Ebola, Zika and other diseases (Bull et al., 2020).

In this study, we performed genome sequencing of SARS-CoV-2 detected in imported cases in Nanjing on the MinION Mk1C platform, focusing on the genomic features, variation analysis, phylogenetic analysis, and evolutionary patterns of the SARS-CoV-2. The evolution and variation characteristics of SARS-CoV-2 imported from abroad in Nanjing were continuously monitored.

## Materials and Methods

### Sampling and Viral RNA Extraction

All specimens were throat swabs originating from imported infections in Nanjing during 2020–2021. The specimens were stored at 4°C and tested on the same day. Total RNA was collected in a biosafety level 2 (BSL 2) laboratory at the Nanjing Municipal Center for Disease Control and Prevention with personal protection equipment for biosafety level 3 laboratory. Specimens underwent total RNA extraction using the Nucleic acid extraction and purification kit on an automated extraction instrument (bioPerfectus technologies, SSNP-3000A, China). The nucleic acid was extracted according to the instructions and stored at 4°C for later use.

### Quantitative Real-Time PCR Analysis

2019-nCoV Nucleic acid detection Kits (qRT-PCR) (bioPerfectus technologies and Daan Gene, China) were used to test SARS-CoV-2. Based on one-step RT-PCR, the kits selected ORF1ab and N genes of SARS-CoV-2 as amplification target regions. Ribonuclease P (RNP) was used as an internal reference gene. The 20 μL reaction mixture consisted of 5 μL RNA sample and 15 μL freshly prepared mixture. Quantitative reverse-transcriptase polymerase chain reaction (qRT-PCR) was performed in an QuantStudio™ 5 Real-Time PCR Instrument (96-Well 0.2 mL Block) (Applied Biosystems, United States) and the bioPerfectus technologies conditions are as follows: 50°C for 10 min, 97°C for 1 min followed by 45 cycles of 97°C for 5 s, 58°C for 30 s (Daan Gene: 50°C for 15 min, 95°C for 15 min followed by 45 cycles of 94°C for 15 s, 55°C for 45 s). All the experimental data were obtained from three biological replicates. Positive or suspected positive samples will be sequenced.

### Oxford-Nanopore Sequencing of SARS-CoV-2

cDNA was synthesized by the use of Target Capture Kit for SARS-CoV-2 Whole Genome (Baiyi Technology Co., Ltd., China) from viral RNA extracts, which contains random hexamers. Whole genome sequences of SARS-CoV-2 was obtained to enrich cDNA by primer pool 1 and primer pool 2 with 2.5 μL of cDNA per reaction. Amplification was performed with Veriti^®^ 96-Well Thermal Cycler (Applied Biosystems, United States). DNA amplicons were cleaned using AMPure XP beads (Beckman coulter, United States), quantified using Qubit 3.0 Fluorometer (Invitrogen, United States) with Qubit™ dsDNA HS Assay Kit (Invitrogen, United States). The construction of DNA library consists of three parts: DNA Repair, Native barcode ligation and Adapter ligation and clean-up. Enzymes of NEB (3.5 μL NEBNext FFPE DNA Repair Buffer, 2 μL NEBNext FFPE DNA Repair Mix, 3.5 μL Ultra II End Prep reaction buffer and 3 μL Ultra II end Prep enzyme Mix) were used for DNA (48 μL) repair and end-prep. 300 ng end-prepped DNA (12.5 μL), Native Barcode (2.5 μL) and Blunt/TA Ligase Master Mix (15 μL) were performed on Native barcode ligation using the ONT Native Barcoding Expansion 1-12 kit (EXP-NBD104). AMPure XP beads and 70% alcohol were used to purify DNA, and quantify with Qubit 3.0 Fluorometer. Add the sample with equal molar volume connected to barcode into a new 1.5 ml centrifuge tube, with a total volume of not less than 400 ng and a volume of 65 μL. 400 ng pooled barcoded sample (65 μL), Adapter Mix II (AMII) (5 μL), NEBNext Quick Ligation Reaction Buffer (5×) (20 μL) and Quick T4 DNA Ligase (10 μL) were used to ligate adapter with the ONT Ligation Sequencing kit (SQK-LSK109). Finally, Sequencing Buffer (SQB) (37.5 μL), Loading Beads (LB) (25.5 μL) and DNA library (12 μL) were loaded onto the SpotON flow cell. Sequencing was performed on the MinION Mk1C (Oxford Nanopore, United Kingdom) platform. The run was terminated and the flow-cell washed using the ONT Flow Cell Wash kit (EXP-WSH004), allowing re-use in subsequent sequencings. All kits were used according to the manufacturer’s protocol.

### Variation Analysis of SARS-CoV-2

In this study, we used the mined data to analyze the genomic variability of SARS-CoV-2 in order to identify its conserved domains and mutational patterns. The sequence of SARS-CoV2 reference genome Wuhan-Hu-1 (GenBank Accession No: MN908947.3) was retrieved from the National Center for Biotechnology Information (NCBI) database and the nucleotide and amino acid positions of each protein in the target SARS-CoV2 genome were located. Conversion tool guppy (V5.0.11^2^) was used to process the original fast5 files to generate FASTQ files, and then use fastp (V0.20.1^3^) (Chen et al., 2018) to conduct quality control on the samples. NanoFilt (V2.8.0^4^) (Coster et al., 2018) was used to filter sequencing data which less than 200 bp and more than 700 bp in length. Minimap2 (V2.17^5^) (Li, 2018) was used to compare our genome data with Wuhan-Hu-1. Medaka (V1.4.3^6^) and snpEff (V5.0^7^) (Cingolani et al., 2012) were used to detect the nucleotide and protein mutation sites, and finally the mutation frequency was calculated. In order to better explore the impact of the genome variation, we selected the variation sites with a variation frequency greater than 0.05. We mainly focus on the variation of gene coding region and the sites causing amino acid changes. In order to further study the effect of variation on Pango lineages, infectivity and virulence, we counted all variant sites.

### Phylogenetic Analysis

We collected 4,175,019 genome sequences and related information of SARS-CoV-2 from the GISAID database (^8^ updated on October 09, 2021) (Khare et al., 2021). Duplicates and low-quality sequences (>5% of the N region) were deleted. Due to the large number of genome sequences, we constructed a minimum spanning tree using 3,270 samples (42 samples imported from Nanjing, 1,228 GISAID samples sequenced in China and 2,000 samples randomly selected according to the national proportion). To understand the distribution and development of Variants of Interest (VOIs) and Variants of Concern (VOCs) in different countries, Alpha (B.1.1.7, Q.1-Q.8), Beta (B.1.351, B.1.351.1-B.1.351.5), Gamma (P.1, P.1.1-P.1.12), Delta (B.1.617.2, AY.3-AY.37, Others) and Lambda (C37, C37.1) (*n* = 1,500) were selected to constructed minimum spanning trees, respectively. All sequences are sorted by name, as well as by country and time. All data were randomly obtained by software seqtk^9^.

Snippy (V4.6.0^10^) was used to obtain the mutation site, and the adjacency matrix was obtained. The differences between samples were analyzed based on the adjacency matrix, and clustering analysis was carried out according to euclidean distance through the sns.clustermap (V0.11.1) in Python. The alignment was input into ModelFinder Plus to assess the best fit substitution model (GTR+F+R6). In order to further explore the evolutionary relationship between samples, the Bayesian phylogenetic tree was generated with IQ-TREE (V2.1.2^11^) (Nguyen et al., 2015; Kalyaanamoorthy et al., 2017)using the GTR+F+R6 substitution model. Boot-strap support was established through 1,000 iterations for Ultra-Fast Bootstrapping (UFBoot), and the iTOL^12^ was used for exhibition. In order to explore the evolutionary relationship and trend of Pango lineages in different countries, the GrapeTree (V1.5.0^13^) (Zhou et al., 2018) was used to build a minimum spanning tree, and Pango lineage and country were added for analysis.

## Results

### Genetic Characteristics of SARS-CoV-2

Nanjing (118°22′E∼119°14′E, 31°14′N∼32°23′N) (Sun et al., 2020) is the capital of Jiangsu Province, China. Nanjing covers an area of 6,587.02 km^2^ and has a population of approximately 9.31 million (Nanjing Municipal Bureau Statistics). Nanjing is an important transportation hub city in Eastern China, with a total passenger transport volume of 113.745 million in 2020 (Nanjing Municipal Bureau of Industry and Information Technology). From 2020 to 2021, a total of 42 confirmed cases of SARS-COV-2 infection were collected from inbound travelers using Quantitative Real-Time PCR (qRT-PCR). We deeply sequenced the 42 positive samples using Oxford-Nanopore Sequencing platform. The sequencing time was adjusted according to the number of samples. The genomes were obtained using a conventional workflow, with an average sequencing depth of 3453.34×. For 42 samples, the whole genome sequences of 41 samples were basically obtained, with a coverage depth of 97.71–99.94%. The sequencing data of 200324-8 is only 3.1 Mb, 5,712 reads, and the sequencing depth is 83.11, but the genome coverage still reached at 91.25%. For 41 sample, 34.32 to 713.04 Mb (mean 265.94 Mb, median 217.59 Mb) of high-quality data were saved, with reads per sample ranging from 60,029 to 1,022,003 (mean 452,648.00, median 387,513.00). In particular, some samples with high Ct values, such as 210316-1 and 210518-8, also obtained genomes with integrity between 99.07 and 99.93%. A total of 16 lineages were identified by whole-genome sequencing in which the dominant lineages were: B.1.1 (*n* = 10), B.1 (*n* = 6), B.1.1.7 (*n* = 5), B.1.617.2 (*n* = 3), B.1.177 (*n* = 2), B.1.351 (*n* = 2), B.40 (*n* = 2), P.1 (*n* = 2) and P.3 (*n* = 2). The sequencing information of samples, such as strain number, source, Ct value, sequencing depth, coverage, Pango lineages, accession numbers, etc., are shown in Supplementary Table 1. All the genome sequences of SARS-CoV-2 sequenced in this study have been deposited in GenBank under the accession numbers: OL989057 – OL989098.

### Mutations Identified in the Sequenced SARS-CoV-2 Genomes

**FIGURE 1:**
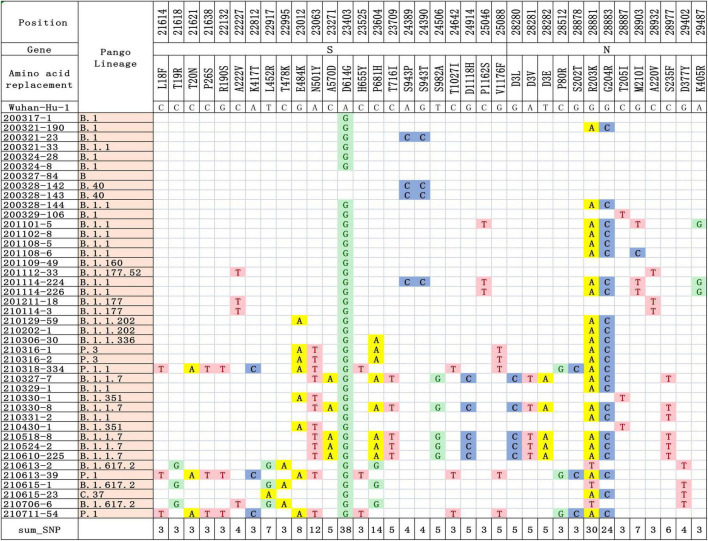
Differences in nucleotide and amino acid substitution in the genomes of SARS-CoV-2 in this study. The sequence of reference genome Wuhan-Hu-1 (GenBank Accession No: MN908947.3) was compared to locate the nucleotide and amino acid positions in the target SARS-CoV-2 genome.

**FIGURE 2:**
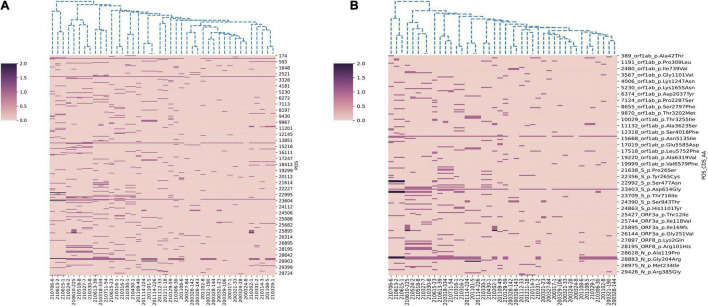
Hierarchical clustering of nucleotide and amino acid mutations in 42 SARS-CoV-2 genomes. **(A)** The nucleotide mutations sites. **(B)** The amino acid mutations sites. The reference genome is Wuhan-Hu-1 (GenBank Accession No: MN908947.3). A color scale indicates the numbers of mutation sites in each genome, from lowest (pink) to highest (purple).

In this study, we performed genome sequencing of SARS-CoV-2 to unveil the genomic variation. The results showed that there are a total of 328 nucleotide mutation sites, of which 205 were non-synonymous mutations and 112 were synonymous mutations. To understand these variants, we mapped polymorphic sites of the genomes, estimated host virus diversity in each sample, and generated a list of intra-host single-nucleotide variants (iSNVs). Sequencing results demonstrated that there were numerous single nucleotide polymorphisms (SNPs) in the open reading frames 1ab (ORF1ab), S, ORF3a and N genes (Figure 1 and Supplementary Table 2). These mutation hotspots may be of keen interest in the adaptability of SARS-CoV-2 to the human host. While, the number of SNPs in the E, M, ORF6, ORF7a, ORF8, and ORF10 genes were null or limited. Limited changes in these proteins may indicate that they have conserved functions, which are necessary for virus propagation. In addition, according to SNPs, SARS-CoV-2 from the same country in different Pango lineages had closer mutation sites. In B.1.1.7 variant, the nt3177 of SARS-CoV-2 from Argentina entered at different times changed from C to T, while the same site of SARS-CoV-2 from Italy remained C. The nt18950 of SARS-CoV-2 imported from Italy at different times changed from A to G. while the one from Argentina remained A (Supplementary Table 2). Except for a few lineages (200324-8, 200327-84, 200328-142, and 200328-143), most of the sequences had a stable nucleotide variation at site 241, 3037, 14408, and 23403 (Figure 2A). 201114-224 and 201114-226 were close contacts of case 201101-5. Their genome sequences were highly similar and clustered into the same branch of the phylogenetic tree. 200328-142 and 200328-143 are a couple, and their genome sequences are also clustered. To other sequences except 200327-84, 200328-142 and 200328-143, the D614G amino acid change in the S protein occurred by an A23403G nucleotide mutation, which confirmed to be associated with greater infectivity (Figure 2B).

### Phylogenetic Analysis of the SARS-CoV-2 Genomes

**FIGURE 3:**
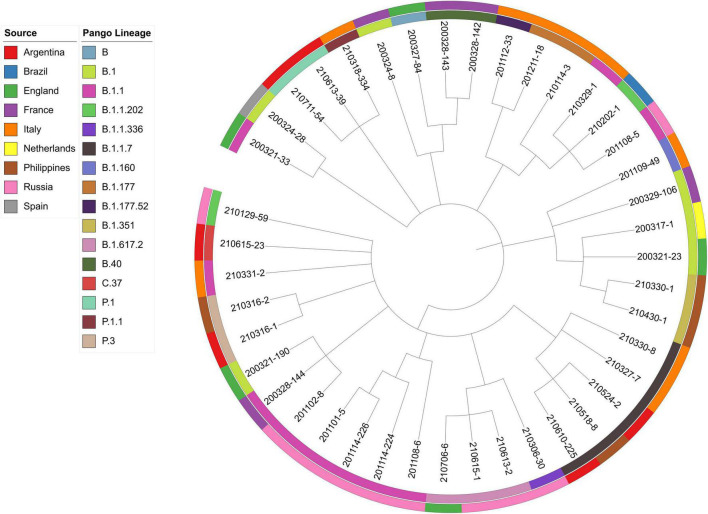
Phylogenetic analysis of 42 samples imported from abroad in Nanjing. The sources and lineages are marked with different colors. The following data are shown (from outer to inner): sources, Pango lineages and samples.

**FIGURE 4:**
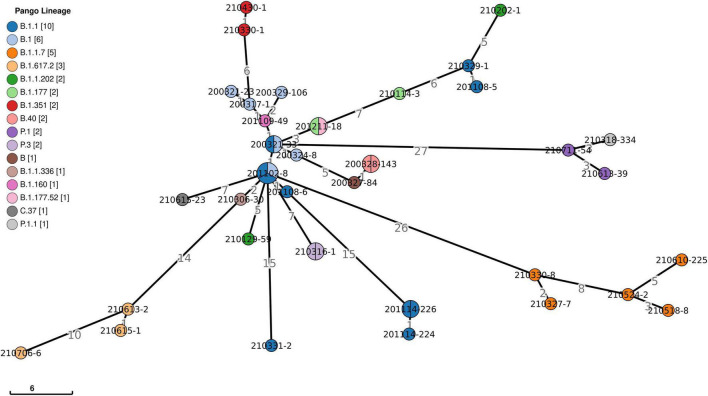
Haplotype network analysis using genome-wide single-nucleotide variations of 42 SARS-CoV-2. It represents genetic relationship or distances between 42 isolates of SARS-CoV-2. The colors represent different lineages. The numbers show genetic distances in SNP differences. SNP, single nucleotide polymorphism.

**FIGURE 5:**
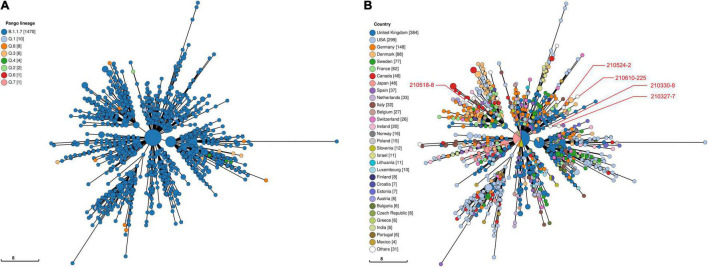
Haplotype network analysis using genome-wide single-nucleotide variations of VOC Alpha in the world. The VOC Alpha were compared with GISAID-available SARS-CoV-2 genomes (*n* = 1,505, updated on October 09, 2021). **(A)** The Pango lineages of VOC Alpha. The lineages are marked with different colors. **(B)** The countries distribution of VOC Alpha. The colors represent different countries. Scale lengths represent the genetic distance.

**FIGURE 6:**
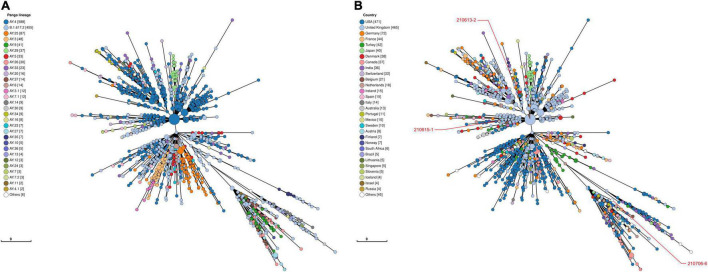
Haplotype network analysis using genome-wide single-nucleotide variations of VOC Delta in the world. The VOC Delta were compared with GISAID-available SARS-CoV-2 genomes (*n* = 1,503, updated on October 09, 2021). **(A)** The Pango lineages of VOC Delta. The lineages are marked with different colors. **(B)** The countries distribution of VOC Delta. The colors represent different countries. Scale lengths represent the genetic distance.

Viral phylogeny is based on the consensus sequence assembled of each sample, and the branching indicates the evolutionary differences in the consensus sequence between samples. All 42 genomes were selected for phylogenetic analysis of SARS-CoV-2 from imported cases in Nanjing (Figure 3). The viral lineage distribution of the imported cases represented some of the SARS-CoV-2 genetic lineages that were circulating worldwide at that time. Phylogenetic analysis showed that the sequences of the same Pango lineages from the same country were more closely related. Haplotype network analysis was performed on the obtained genomic sequences using genome-wide single-nucleotide variation (SNV) (Figure 4). The results showed the genetic distance of 42 samples, and the samples of the same lineage were clustered. Whole-genome sequences of 42 samples were compared with 3,228 SARS-CoV-2 genomes provided by the publicly accessible Global Initiative on Sharing All Influenza Data (GISAID) database (1,228 GISAID samples sequenced in China and 2,000 samples randomly selected according to the national proportion) (*n* = 3,270), and the minimum spanning tree was constructed (Supplementary Figure 1). The same Pango lineage was clustered, which was consistent with their input country. The minimum spanning trees of Alpha, Beta, Gamma, Delta and Lambda variants were constructed, respectively, to visually display the transmission and rules of different lineages in different countries. B.1.1.7 variant was first discovered and transmitted in the United Kingdom (September-2020), while Q.3 and Q.8 were mostly found in the United States and Q.4 in France (Figure 5). B.1.351 variant originated in South Africa (May-2020) and quickly spread to other countries, clustering in the United States, Sweden, Philippines, Luxembourg, Finland and other countries, while B.1.351.5 variant was distributed in Spain and B.1.351.3 variant was in Bangladesh (Supplementary Figure 2). P.1 variant was first identified in Brazil (November-2020), and has since become predominant in the United States and clustered in Canada, Chile, and other countries. P.1.10 variant was mostly distributed in the United States, while P.1.7 and P.1.8 variants were more common in Brazil (Supplementary Figure 3). B.1.617.2 variant was transmitted in India (October-2020), while AY.4 variant was mainly prevalent in the United Kingdom, with an increasing proportion of the Delta variants. AY.25, AY.3, and AY.3.1 variants were clustered, respectively, and were mainly distributed in the United States. AY.29 variant was mainly focused on Japan. The Delta variants are now rapidly becoming the dominant pandemic virus strain globally (Figure 6). C.37 variant was first discovered in Peru (December-2020) and later spread to many countries. And C.37.1 Mostly distributed in Spain, Switzerland, and the United States (Supplementary Figure 4).

## Discussion

SARS-CoV-2 is a highly infectious and pathogenic virus that has deprived millions of lives globally since outbreak in December 2019. Finding the source of infection is one of the most important tasks in the prevention and treatment of viruses, and tracing the virus genome is an important method to find the source and path of infection. Therefore, it is necessary to explore the whole genome sequencing (WGS) data of SARS-CoV-2 to understand its variation characteristics. In this study, 42 SARS-CoV-2 genomes were selected to systematically analyze the nucleotide and amino acid variation characteristics of SARS-COV-2 imported from abroad to Nanjing during 2020–2021, which may have contributed to its virulence, pathogenicity and transmission in the region. The research suggested that the evolution and variation characteristics of SARS-CoV-2 imported from Nanjing should be monitored continuously. This study will provide a theoretical basis for epidemic source tracing and precise prevention and control.

The pathogen of the COVID-19 pandemic, SARS-CoV-2, is steadily mutating amid continued human-to-human transmission. SARS-CoV-2 has accumulated mutations in viral genes encoding the ORF1ab, ORF3a, N and S proteins, among which mutations in the S protein are particularly important because the S protein is key for the first step of viral transmission. D614G, in the S protein of SARS-CoV-2, which has emerged as a predominant clade in Europe and was spreading worldwide (Isabel et al., 2020). Ozono et al. (2021) concluded that the D614G mutation enhanced binding affinity for ACE2 while maintaining neutralization susceptibility, increasing cell entry no infiuence on the antigenicity of the S protein. Zhou et al. (2021) demonstrated that D614G has enhanced binding to the ACE2, increased replication in human bronchial and nasal airway epithelial cultures and in a human ACE2 knock-in mouse model, and increased replication and transmission in SARS-CoV-2-infected hamster and ferret models. Their data explained the global predominance of D614G mutants (Zhou et al., 2021). After a few months of transmission, this variant replaced the previous SARS-CoV-2 as the dominant circulating variant. In this study, we analyzed genomic variability of SARS-CoV-2 imported to Nanjing to identify its conserved domain and mutational patterns. D614G mutation can be found in most of our genome sequences, which is consistent with the global variation trend of SARS-CoV-2. The D614G mutation was the beginning of the SARS-CoV-2 mutation. There were some persistent mutations in virus sequences from different countries, suggesting similarities in the evolution of SARS-CoV-2 across the world. Continuous monitoring of genetic variation in SARS-CoV-2 is necessary, with a focus on key mutation sites. In order to better identify the SARS-CoV-2 types in various countries, Zhang et al. (2020) further classified L-lineage into the European branch of L-lineage according to the whole genome sequence of the virus detected in imported cases in China. The characteristic substitution sites were nt241 (C→T), nt3037 (C→T), nt14408 (C→T), nt23403 (A→G) (Zhang et al., 2020). At the same time, we found synonymous mutations at nucleotide sites 241 and 3,037 in most genomes. These results indicate that multiple synonymous substitutions occur in highly conserved genes during the evolution of viruses, and the locations of these substitutions may affect the viability of viruses. Although it is not as significant as non-synonymous mutations, further strengthening the monitoring of these sites may help to elucidate the genomic characteristics of SARS-CoV-2, and provide guidance for mutation studies in the future.

Following the D614G mutation, several novel SARS-CoV-2 variants of concern (VOCs) carrying the D614G mutation have emerged rapidly and are associated with large-scale infections worldwide. New lineages of SARS-CoV-2 have attracted more attention worldwide because of their higher transmissibility, risk of serious consequences, and/or escape from neutralizing antibodies. VOC Alpha (B.1.1.7 Lineage, etc.), the first SARS-CoV-2 VOC, has been shown to be 43–90% higher reproduction rate and 75% more infectious than the earlier strains, becoming the dominant variant in the United Kingdom and endemic worldwide (Davies et al., 2021; Leung et al., 2021). And the transmission rate of B.1.1.7 increased with age and viral load (Lyngse et al., 2021). It was found that the mutations in the B.1.1.7 S protein had multiple structural effects and could significantly improve its viral fusion activity and infectivity (Xia et al., 2021). As SARS-CoV-2 continues to circulate globally, more SARS-CoV-2 variants have emerged including VOC Beta (B.1.351 lineage, etc.) and VOC Gamma (P.1 lineage, etc.) that were first detected in South Africa and Brazil, respectively. Preliminary modeling suggested that the B.1.351 variant could be approximately 50% more transmissible than early SARS-CoV-2 strains (Tegally et al., 2021). P.1 may be 1.7- to 2.4-fold more transmissible and can evade 21–46% of protective immunity elicited by previous infection with non-P.1 lineages (Faria et al., 2021). Subsequently, the Delta (B.1.617.2 Lineage, etc.) variant spread across India, leading to a rapid increase in COVID-19 cases in many countries around the world, outcompeting pre-existing lineages including B.1.1.7 (Alpha) (Mlcochova et al., 2021). These variants evade neutralizing antibodies and is considered to be more infectious and pathogenic (Liu et al., 2021a,b). We selected VOC Alpha, Beta, Gamma and Delta lineages to construct minimum spanning trees (Figures 5, 6 and Supplementary Figures 2, 3) to visually display the genetic relationships and distances among mutants distributed in different countries. Mutations in different countries may differ due to host selection pressures. Genome sequence has obvious regional characteristics, and some mutation sites in the same country are consistent. These results indicate that although some mutations are inherent in the evolution of SARS-CoV-2, some mutations may be the result of adaptation of the virus to specific countries, social economic factors, medical care, sex-age ratio and natural environment. Of the B.1.1.7 variants imported to Nanjing, two were from Italy, one was from Philippines and two were from Argentina in March, May and June 2021, all during the VOC Alpha pandemic. Two cases infected with B.1.351 variant from the Philippines were imported into Nanjing in March and April 2021, respectively. Two cases infected with P.1 variant came from Argentina and were imported in June and July 2021, respectively. Three cases infected with B.1.617.2 variant from Russia and England were imported in June and July 2021, respectively. We observed that at least two genomic sequences in the B.1.1.7 lineage shared the same non-synonymous substitutions at nt3177 (C→T), nt4919 (A→G), nt6936 (C→T), nt14382 (T→C), nt15216 (C→T), nt15668 (A→T), nt18950 (A→G), nt20844 (C→T), nt24374 (C→T), nt25785 (G→T), and nt25855 (G→T), excluding the sites where all the sequences were mutated. The substitutions of all sequences were nt241 (C→T), nt913 (C→T), nt3037 (C→T), nt3267 (C→T), nt5388 (C→A), nt5986 (C→T), nt6954 (T→C), nt14408 (C→T), nt14676 (C→T), nt15279 (C→T), nt16176 (T→C), nt23063 (A→T), nt23271 (C→A), nt23403 (A→G), nt23604 (C→A), nt23709 (C→T), nt24506 (T→G), nt24914 (G→C), nt27972 (C→T), nt28048 (G→T), nt28111 (A→G), nt28280 (G→C), nt28881 (G→A), nt28882 (G→A), nt28883 (G→C), and nt28977 (C→T). In the genome sequences of B.1.351, the sites containing non-synonymous substitutions were nt1059 (C→T), nt5230 (G→T), nt10323 (A→G), nt14408 (C→T), nt15925 (C→T), nt21641 (G→T), nt22206 (A→G), nt22813 (G→T), nt23012 (G→A), nt23063 (A→T), nt23403 (A→G), nt23664 (C→T), nt25563 (G→T), nt26456 (C→T), nt28887 (C→T). The sites of synonymous substitutions were nt3037 (C→T), nt4213 (T→C), nt11812 (C→A), nt23764 (A→T), nt28642 (T→C). In the genome sequences of P.1, the sites containing non-synonymous substitutions were nt3828 (C→T), nt5648 (A→C), nt14408 (C→T), nt17259 (G→T), nt21614 (C→T), nt21621 (C→A), nt21638 (C→T), nt22132 (G→T), nt22812 (A→C), nt23012 (G→A), nt23063 (A→T), nt23403 (A→G), nt23525 (C→T), nt24642 (C→T), nt25088 (G→T), nt26149 (T→C), nt28167 (G→A), nt28512 (C→G), nt28878 (G→C), nt28881 (G→A), nt28883 (G→C). The sites of synonymous substitution were nt733 (T→C), nt2596 (A→G), nt2749 (C→T), nt3037 (C→T), nt6319 (A→G), nt6613 (A→G), nt12778 (C→T), nt13860 (C→T), nt18129 (C→T), nt28877 (A→T), nt28882 (G→A). In addition, there are at least two genome sequences in the B.1.617.2 lineage had the same non-synonymous substitutions at nt4181 (G→T), nt6402 (C→T), nt7124 (C→T), nt9053 (G→T), nt10029 (C→T), nt11201 (A→G) and nt28916 (G→T). The substitutions of all sequences were nt210 (G→T), nt241 (C→T), nt3037 (C→T), nt14408 (C→T), nt15451 (G→A), nt16466 (C→T), nt21618 (C→G), nt22917 (T→G), nt22995 (C→A), nt23403 (A→G), nt23604 (C→G), nt25469 (C→T), nt26767 (T→C), nt28881 (G→T), and nt29402 (G→T). During the evolution of SARS-CoV-2, it is worth paying attention to the clustered mutations, which may be associated with the further epidemic of the virus, and the relatively new stable mutations may indicate the direction of the virus evolution. For strains that have formed stable Lineages, the detection of specific substitutions is helpful for virus tracing. Whether and how these substitutions play a role in infectivity and immunogenicity of SARS-CoV-2 remains to be further studied.

There are some limitations in this study: Firstly, the samples of this study are from the imported infection cases in Nanjing, so the results and its application are limited. Secondly, our research samples are not big enough, so that we cannot get a full picture of the spectrum of SARS-CoV-2. Thirdly, since no imported cases of Omicron infection have been found in Nanjing, our study does not include Omicron variant, which is a bit insufficient compared with current concern.

In early 2020, evolutionary biologist Jesse Bloom looked into the future of SARS-CoV-2, predicting that instead of eradicating the new pathogen, it would become the fifth coronavirus to persist permanently in humans. Scientists expect SARS-CoV-2 will eventually evolve in a more predictable way and become like other respiratory viruses, but when that transition will happen and what kind of infection it might resemble is unclear (Callaway, 2021). With the arrival of winter, the health risks facing the global population and the risk of the spread of outbreaks are very high. The current global control capacity is insufficient, and there are huge disparities in affordability, post-infection treatment and vaccination in different countries. Therefore, we need to strengthen international cooperation, build a global epidemic prevention and control system, improve governance capacity, and jointly face the huge challenges brought by SARS-CoV-2. In addition, a variety of SARS-CoV-2 variants followed, with different immunogenicity and infectivity. The epidemic situation is still grim and complex, and the prevention and control of the epidemic cannot be slack. We need to closely track the impact of mutations in the existing mutations and watch out for new ones to strengthen global surveillance of viral mutations, focusing on the impact of key mutation sites of SARS-CoV-2 on viral transmission, clinical manifestations and severity. Quarantining and monitoring of imported infections should be carried out to prevent large-scale local transmission. In the future, normalized epidemic prevention and control measures need to be optimized and improved to make prevention and control policy more scientific and accurate.

## Data Availability Statement

The datasets presented in this study can be found in the NCBI at: https://www.ncbi.nlm.nih.gov/nuccore, accession numbers OL989057–OL989098.

## Author Contributions

MH, NaZ, and HF conceived and designed the experiments. NiZ, XqD, XxD, DX, XM, YY, and HG performed the experiments. XX and NiZ analyzed the data. LM contributed reagents, materials, and analysis tools. NiZ wrote the manuscript. JD reviewed and edited. All authors contributed to the article and approved the submitted version.

## Conflict of Interest

XX and LM are employed by Hangzhou Baocheng Biotechnology Co., Ltd. The remaining authors declare that the research was conducted in the absence of any commercial or financial relationships that could be construed as a potential conflict of interest.

## Publisher’s Note

All claims expressed in this article are solely those of the authors and do not necessarily represent those of their affiliated organizations, or those of the publisher, the editors and the reviewers. Any product that may be evaluated in this article, or claim that may be made by its manufacturer, is not guaranteed or endorsed by the publisher.

## Footnotes

1
https://covid19.who.int/

2
https://mirror.oxfordnanoportal.com/software/analysis/ont-guppy_5.0.11_linux64.tar.gz

3
https://github.com/OpenGene/fastp

4
https://github.com/wdecoster/nanofilt

5
https://github.com/lh3/minimap2

6
https://github.com/nanoporetech/medaka

7
https://pcingola.github.io/SnpEff/

8
http://gisaid.org/

9
https://github.com/lh3/seqtk

10
https://github.com/tseemann/snippy

11
https://github.com/iqtree/iqtree2

12
https://itol.embl.de/

13
https://achtman-lab.github.io/GrapeTree/MSTree_holder.html
