# Supplementary material for: Mutations and Phylogenetic Analyses of SARS-CoV-2 Among Imported COVID-19 From Abroad in Nanjing, China
Source: Front Microbiol. 2022 Mar 17;13:851323. doi: 10.3389/fmicb.2022.851323 (PMC8969601; doi:10.3389/fmicb.2022.851323)
Supplement: Supplementary file 1 [file Table_1.DOCX]

***Supplementary Material***

**
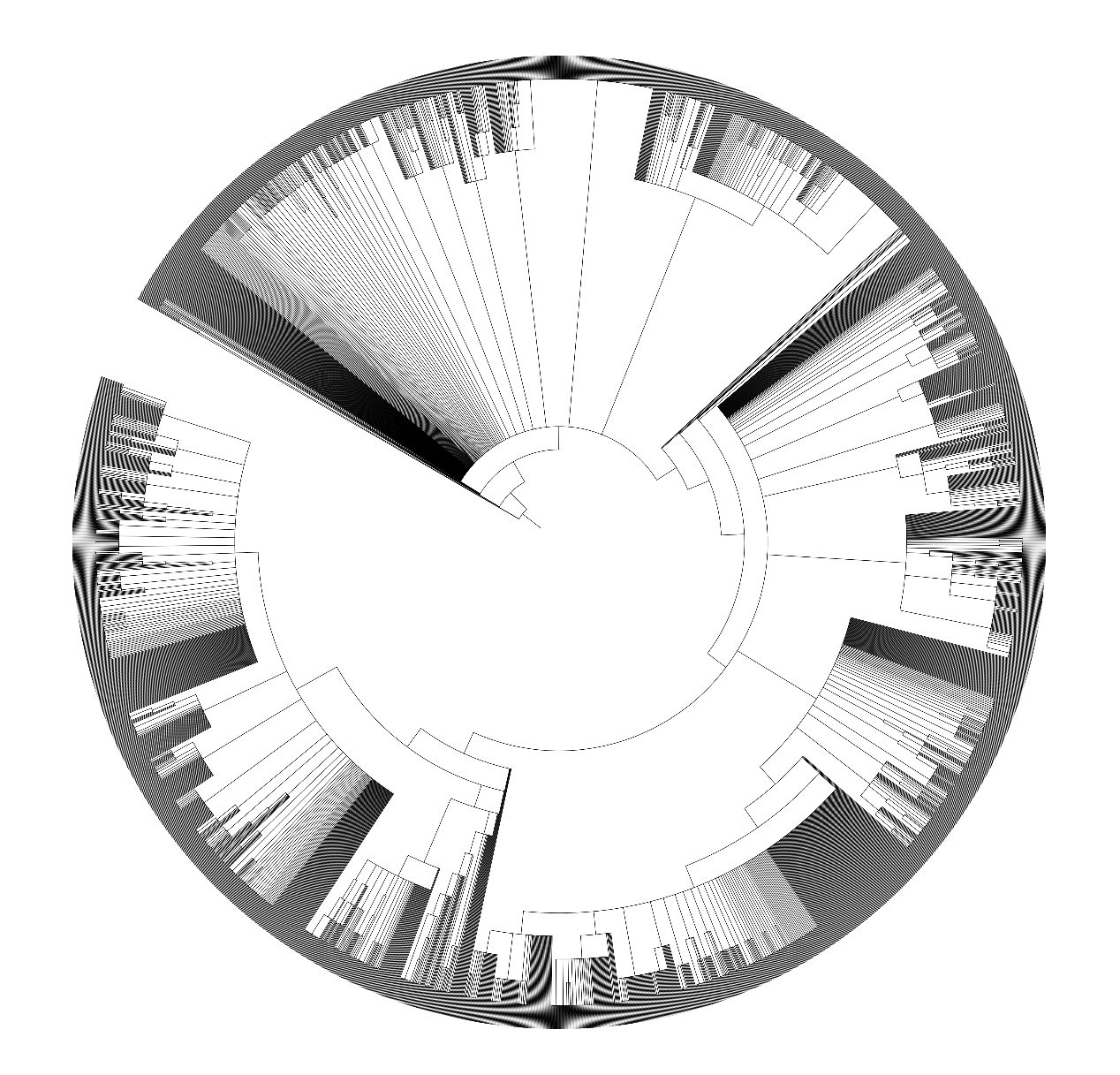
Supplementary Figures**

**Figure S1.** A minimum spanning tree was inferred based on the whole-genome sequences of 42 SARS-CoV-2 isolates and GISAID-available SARS-CoV-2 genomes (n = 3270).


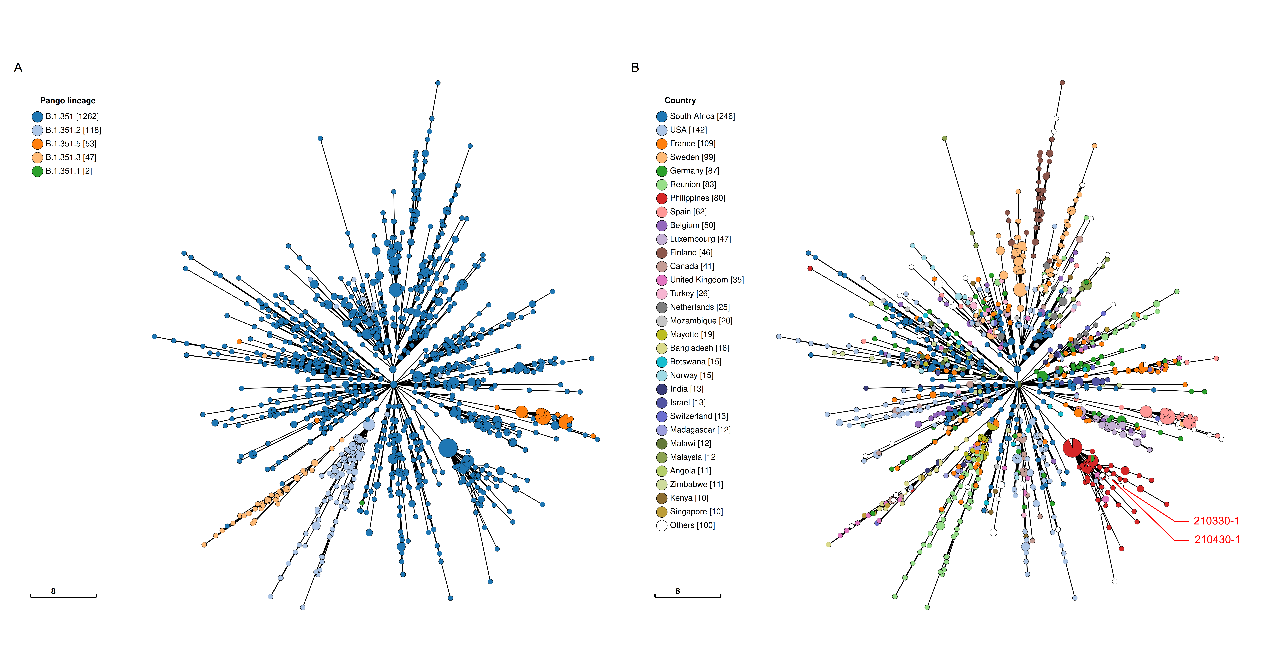


**Figure S2.** Haplotype network analysis using genome-wide single-nucleotide variations of VOC Beta in the world. The VOC Beta were compared with GISAID-available SARS-CoV-2 genomes (n = 1502, updated on October 09, 2021). (A) The Pango lineages of VOC Beta. The lineages are marked with different colors. (B) The countries distribution of VOC Beta. The colors represent different countries. Scale lengths represent the genetic distance.


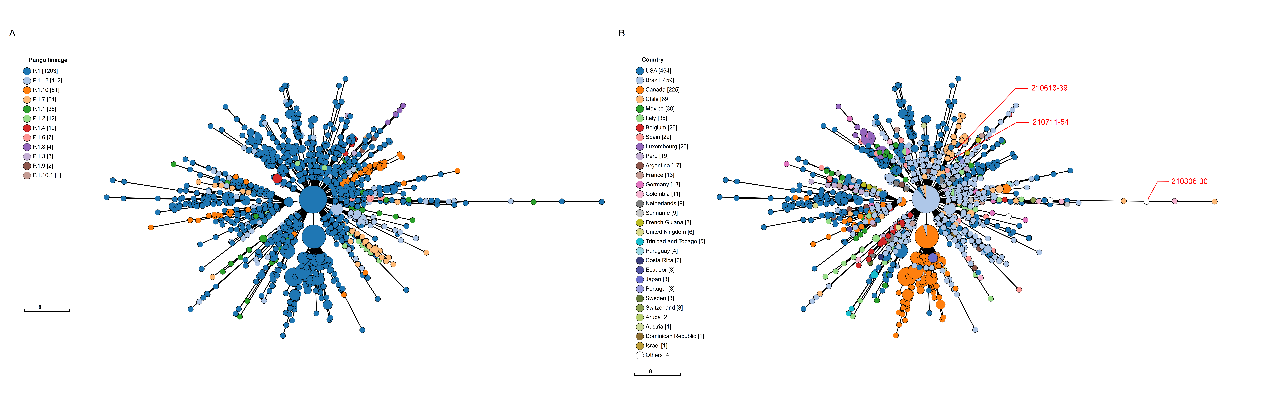


**Figure S3.** Haplotype network analysis using genome-wide single-nucleotide variations of VOC Gamma in the world. The VOC Gamma were compared with GISAID-available SARS-CoV-2 genomes (n = 1502, updated on October 09, 2021). (A) The Pango lineages of VOC Gamma. The lineages are marked with different colors. (B) The countries distribution of VOC Gamma. The colors represent different countries. Scale lengths represent the genetic distance.


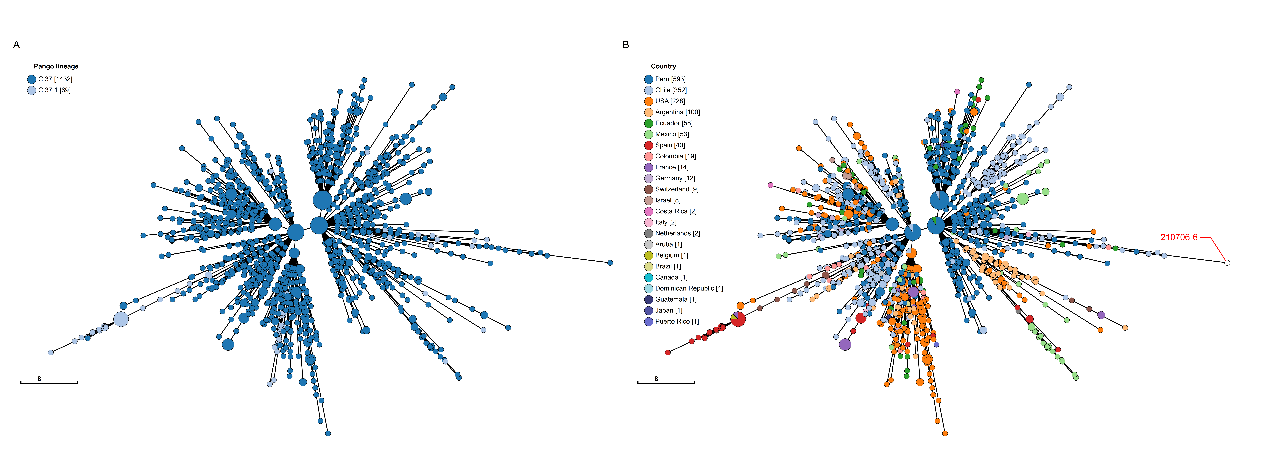


**Figure S4.** Haplotype network analysis using genome-wide single-nucleotide variations of VOC Lambda in the world. The VOC Lambda were compared with GISAID-available SARS-CoV-2 genomes (n = 1501, updated on October 09, 2021). (A) The Pango lineages of VOC Lambda. The lineages are marked with different colors. (B) The countries distribution of VOC Lambda. The colors represent different countries. Scale lengths represent the genetic distance.
